# Development of a Process Technology to Improve the Internal Particle Density and Enhance the Performance of Medical Radiation Shielding Materials

**DOI:** 10.3390/ma18102174

**Published:** 2025-05-08

**Authors:** Seon-Chil Kim

**Affiliations:** Department of Biomedical Engineering, School of Medicine, Keimyung University, 1095 Dalgubeol-daero, Daegu 42601, Republic of Korea; chil@kmu.ac.kr; Tel.: +82-10-4803-7773

**Keywords:** medical radiation, shielding sheet, X-ray, particle mixing, shielding performance

## Abstract

Protective garments for the medical radiation shielding of healthcare professionals must ensure flexibility and shielding performance. As such, process technologies for density enhancement are required when manufacturing shielding sheets to ensure the reproducibility of flexibility and shielding performance. Although previous efforts commonly reduced particle size to minimize porosity, nanoparticle production cost is significant. Therefore, this study aimed to improve the density of the shielding sheet by controlling the spacing between internal particles. The proposed improvement method is based on polydisperse particle packing. Particle sizes can be adjusted using process techniques such as sintering, pressing, and mixing. The study materials used are tungsten and bismuth oxide (eco-friendly alternatives to lead), with polyethylene as the polymer matrix. First, the shielding performance improved by 4% in the sintering process when the tungsten content reached 90 weight percent (wt%). The solvent removal process, used to eliminate the solvent added for polymer utilization, increased the density by 13.18%; however, it was lower than that of the compression process. The shielding performance improved by approximately 10% in the compression molding process when the tungsten content was 90 wt%. This study confirms that optimizing density enhancement strategies for radiation shielding materials can significantly improve shielding performance.

## 1. Introduction

In medical institutions, radiation is used in close proximity to patients and medical staff. Therefore, continuous environmental monitoring and the easy accessibility of shielding tools are crucial to minimizing medical radiation exposure [1,2]. Lead is the primary constituent of conventional shielding materials [3], which is known for its cost-effectiveness and excellent workability; however, environmental pollution concerns have driven the development of eco-friendly alternative materials [4].

X-ray generators, which account for the highest usage of medical radiation, typically operate at tube voltages ranging from 40 to 120 kilovolt peak (kVp). This requires adjustments to shielding material thickness or composition depending on the incident energy intensity [5]. Consequently, factors such as the thickness, weight, and density of shielding materials are directly related to their radiation protection performance [6]. The shielding performance of materials against medical radiation can be enhanced by improving their radiation attenuation effects. This can be achieved by increasing their thickness, using high atomic number materials, or employing heavier materials [7].

For shielding materials used in medical environments, their thickness and weight are critical selection factors [8]. For radiation-protective garments, excessive weight and thickness can restrict user mobility. Conversely, these factors can impose limitations on miniaturization and automation in the case of medical device components. Therefore, process technologies that reduce thickness and weight during the fabrication of shielding materials are crucial for determining the usability of the final shielding tool.

The radiation shielding materials used in medical institutions must primarily have excellent flexibility for practical use, as their primary function is to protect patients and medical personnel [9]. Therefore, the shielding material must exhibit high processability for it to be processed into various defense forms while maintaining reduced thickness [10]. The density of the shielding material is an important process technology for ensuring flexibility at the same shielding performance. Furthermore, these factors depend on the final shielding structure and the materials used [11]. Weight is determined by the physical amount of the shielding material. Conversely, thickness is correlated with density. In same-weight materials, thickness reduction may indicate an increase in density. Thus, density control is a crucial technological aspect in the manufacturing process of shielding materials [12].

Shielding materials can be in the form of films, sheets, and fibers; however, shielding performance varies depending on the dispersion of the material and its compatibility with macromolecules [13]. In addition, the technology used in the manufacturing process influences shielding performance. The key to this process generally involves increasing the radiation interaction probability by uniformly distributing particles within the shielding material or minimizing bubbles and pores, which reduce interparticle gaps [14,15].

Previous studies on shielding sheets have primarily employed mechanical methods, mainly utilizing compaction/pressing and roll milling to mix polymers with metallic materials [16]. While these methods expand the application range of polymers and ensure the flexibility of the sheets, maintaining the reproducibility of shielding performance becomes challenging with increased production volume [17]. Herein, a density control process for shielding sheets is proposed to secure reproducibility in material density in future mass production processes, which can help maintain and improve shielding performance.

In medical institutions, efforts to improve the performance of shielding materials focus on increasing the internal particle density to enhance shielding effectiveness. Although reducing particle size to minimize porosity is commonly employed, the cost of nanoparticle production is significant. Therefore, in this study, we aimed to improve the shielding performance by employing physical processes such as sintering, solvent removal, and compression to reduce porosity while using particles of multiple sizes. This study employs eco-friendly materials and polymer blends, excluding lead. The primary technical approach involves methods to control particle spacing through metal material sintering and combining with polymers. These techniques aim to reduce porosity and enhance density in the shielding materials, ultimately improving shielding performance. Furthermore, the ratio of composite materials and process standardization are the most important factors that can help increase the product density during material preparation, mixing, and molding processes [18].

Enhancing the compatibility between shielding materials and polymers is essential for optimizing dispersion. This study aims to present methods for improving the density of shielding materials through particle packing optimization, sintering, or solvent evaporation during the mixing process. The improved density is ultimately evaluated by comparing and validating its effect on the shielding performance of the radiation shielding material.

The sintering of shielding materials involves applying heat to promote interparticle bonding, which effectively reduces porosity within the material [19]. The effect of reducing interparticle spacing increases significantly as porosity decreases, and surface modification can further enhance material dispersion [20]. Enhanced dispersion helps maintain consistent interparticle spacing, prevents material aggregation in localized regions, and minimizes air voids introduced during the mixing process. Particle size is also crucial in achieving optimal dispersion [21]. In addition, solvent selection is crucial to using polymeric binders effectively. The evaporation of the selected solvent can reduce air voids and overall porosity, leading to increased density, which enhances the shielding performance [22].

This study validates the relationship between shielding material density improvement and shielding performance enhancement by refining polymer blending and shielding material processing techniques. Furthermore, an efficient density enhancement process is proposed, which can improve the usability of shielding sheets in medical institutions and increase accessibility to radiation protection solutions.

## 2. Materials and Methods

The initial radiation energy intensity ($I_{0}$) incident on the shielding material differs from the final radiation intensity (I) after transmission through the shielding material. This difference is determined by the thickness of the shielding material (t) and its intrinsic property, the linear attenuation coefficient (*μ*), as described by the following equation [23]:

(1) $$I=I_{0}e^{-\mu t}.$$

The radiation attenuation effect is caused by the interactions between the radiation and the structure of the shielding material [24]. This implies that the higher the number of interactions, the higher the shielding effectiveness. Therefore, increasing the density of the shielding material components can help improve shielding effectiveness. Density is generally defined by the relationship between volume (*υ*) and mass (*m*), as expressed in Equation (2) [25]

(2) $$\rho =\frac{m}{\upsilon }.$$

The radiation penetrating the shielding material is directly related to density and thickness factors, as expressed in Equation (3)

(3) $$I=I_{0}e^{-(\frac{\mu }{\rho }\times \rho t)}.$$

The reduction in radiation as it passes through the shielding material can be described by the mass attenuation coefficient, which can be expressed by the Beer–Lambert law [26], as shown in Equation (4)

(4) $$\mu_{S}=\frac{\mu }{\rho }.$$

Therefore, for composite mixtures such as those in this study, the mass attenuation coefficient can be expressed as the weighted sum of the individual contributions of each element composing the material [27]. Consequently, the overall mass attenuation coefficient of the shielding material is described in Equation (5)

(5) $$\mu_{S}={\left(\frac{\mu }{\rho }\right)}_{mix}=\sum_{i}w_{i}{\left(\frac{\mu }{\rho }\right)}_{i}.$$

Thus, the mass distribution of each element within the material is determined by the initial composition of the composite mixture.

Thus, the arrangement of materials with mass, specifically the control of interparticle spacing through dispersion, is determined by the composition of the composite mixture. In this study, tungsten, bismuth oxide, and polyethylene were selected. A higher number of composite components enhances interactions within the shielding material, thereby directly contributing to shielding effects such as absorption and scattering [28]. In addition, interparticle spacing should be predictably dispersed to optimize these effects. Tungsten, an eco-friendly alternative to lead, was chosen for its extensive applicability and superior shielding efficiency. Bismuth oxide was selected for its high density and cost-effectiveness in complementing tungsten [29,30]. Polyethylene, a polymer material, was chosen as the base material for sheet fabrication owing to its excellent compatibility, light weight, and high flexibility [31].

**Figure 1:**
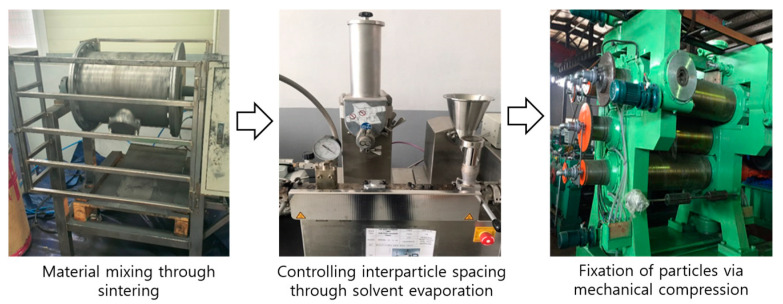
Sintering and mixing, solvent evaporation, and compaction processes during the fabri-cation of the shielding sheet. [Example].

First, when fabricating the shielding sheet, tungsten powder (tungsten, W, 99.9%, 19.3 g/cm^3^, <10 μm, Nangong Xindun Alloy Spraying, Co., Ltd., Xingtai, China) was pulverized for 5 min to adjust the particle size, and subsequently dried in an oven at 60 °C for 24 h, as shown in Figure 1 [32]. Similarly, bismuth oxide (Bi_2_O, 99.9%, 8.9 g/cm^3^, <100 μm, Duksan General Science, Seoul, Republic of Korea) was pulverized for 5 min and dried in an oven at 60 °C for 24 h, following the same procedure as tungsten powder, and was subsequently used. The polymer polyethylene (PE, P-8241A, Mw. 21,000–35,000, Songwon, Busan, Republic of Korea) was processed under the same temperature and drying conditions as those applied to the shielding materials. Tetrahydrofuran (THF, 99.5%, Daejung, Siheung, Republic of Korea) was used as the solvent for polymer dissolution, while chloroform (95%, Duksan, Soul, Republic of Korea) was employed as a poor solvent to control the evaporation rate. All solvents were used without any special pretreatment. THF was stirred at approximately 10 weight percent (wt%) to dissolve the polyethylene, and the prepared casting solution was mixed with the material powders. Ultrasonic dispersion (VCX-750, 2021, Sonics, Newtown, CT, USA) was employed to ensure uniform particle dispersion [33]. The material was filtered to remove impurities, degassed, and then subjected to compression molding to maintain consistent shielding performance in the final casting solution [34]. Residual solvents were removed through dehydration and solvent evaporation using a vacuum drying oven (SH-VDO-70NC, 2020, Republic of Korea) at 60 °C for 12 h.

**Figure 2:**
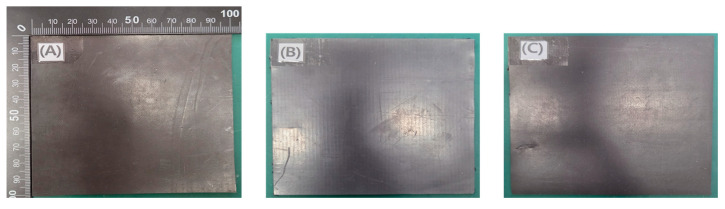
External appearance of the shielding sheets based on the shielding material content: (**A**) Tungsten content (80 wt%); (**B**) Tungsten content (85 wt%); (**C**) Tungsten content (90 wt%).

Compression molding has been widely applied in the fabrication of thermoplastic composites, including polyethylene-based systems [35]. This method was also employed in this study. Using a roll press machine (MR-DG100L, Shenzhen Meirui Zhida Technology, Shenzhen, China), a stepwise compression method was applied to laminate the materials, and the final shielding sheet was fabricated with dimensions of 100 mm × 100 mm × 1 mm, as shown in Figure 2 for three different tungsten contents.

The polyethylene polymer and the shielding materials, tungsten and bismuth oxide, were mixed in varying ratios to evaluate their impact on the shielding performance (Table 1). This was done to analyze differences in the shielding performance depending on the amount of shielding material, and to separately examine the effects of the dispersion characteristics and processing techniques at different mixing ratios. The shielding sheets were fabricated by applying the process technology using three different compositions, 80%, 85%, and 90%, based on tungsten, which exhibited the highest shielding effectiveness. In addition, the density of the fabricated shielding sheet was measured [36], and the density values were calculated using the following equation [37]:

(6) $$\rho =\rho_{w}\frac{m_{1}}{m_{1}-m_{2}},$$

where ρ is density (g/cm^3^), $\rho_{w}$ is the density of distilled water (g/cm^3^) = 0.997989 g/cm^3^, applied at a temperature of 21 °C, $m_{1}$ is the mass of the shielding sheet measured in air (g), and $m_{2}$ is the mass of the shielding sheet measured in distilled water (g).

**Figure 3:**
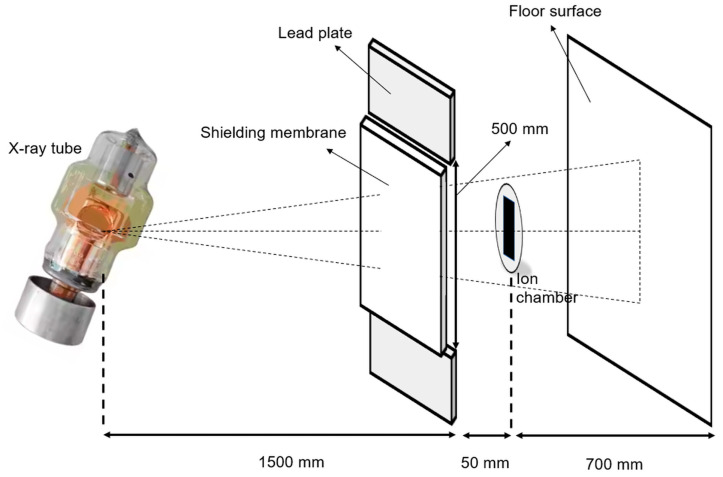
Evaluation of the radiation shielding performance of the shielding sheets.

Thin-section samples of the fabricated shielding sheets were observed using an optical microscope to verify the dispersion of the particles under different process conditions (FESEM; Field Emission Scanning Electron Microscope, Hitachi, Tokyo, Japan S-4800). Through this process, key factors such as particle size, arrangement, and their impact on shielding efficiency were examined. In addition, tensile strength was evaluated, since the physical compression process may reduce the flexibility of the shielding sheet. Tensile strength testing was performed [38], the test method specified in the Korean Industrial Standard for protective aprons for diagnostic X-ray use [39]. Using a universal testing machine (AGX-V, 2010, Shimadzu Corp., Kyoto, Japan), the tests were conducted at a tensile speed of 200 ± 10 mm/min, as shown in Figure 3. Specimens with dimensions of 100 mm × 100 mm were randomly cut from the manufactured sheets for evaluation. For a comparative analysis of shielding performance, an X-ray generator (Toshiba E7239, 150 kV–500 mA, 1999, Tokyo, Japan) was used. In addition, to convert the X-rays used in the experiment into monoenergetic effective energy, the half-value layer (HVL) was calculated based on the attenuation exponential law ($I=I_{0}e^{-\mu x}$): HVL = 0.693/μ [40]. The effective energy was determined using Hubbell’s mass absorption coefficient table, where the effective energy corresponds to the energy with the same HVL value as the measured energy [41]. The X-ray generator operated at tube voltages ranging from 40 to 120 kVp, with an exposure setting of 30 mAs. The dose detector was an ionization chamber dosimeter (TnT 12000, FLUKE Corp., Everett, WA, USA), which was calibrated before use. The experimental method was applied in accordance with the Korean Industrial Standard for testing the lead equivalent of X-ray protective devices [42].

The shielding performance evaluation experiment was set up as shown in Figure 3, and the radiation protection efficiency (RPE) of the fabricated shielding sheets was calculated as described by the following equation [43]:

(7) $$RPE=(1-\frac{W}{W_{0}})\times 100,$$

where W  represents the measured radiation dose when the shielding sheet is placed between the X-ray beam and detector, and $W_{0}$ represents the measured radiation dose when no shielding sheet is present between the X-ray beam and detector.

## 3. Results

The particle morphology was modified through material sintering, interparticle distance was reduced via solvent evaporation of the polymer matrix, and particle arrangement was controlled through compression during the forming stage to improve the material density by controlling the spacing between particles within the shielding structure. The quantitative changes in density and the corresponding improvements in shielding performance were comparatively evaluated.

First, changes in the physical properties of the shielding sheets resulting from the sintering process were analyzed. The shielding sheets were fabricated in three different compositions based on the tungsten content: 80 wt%, 85 wt%, and 90 wt%. Each sheet had a thickness of 0.3 mm, with slight variations of 0.3 ± 0.05 mm owing to the compression and dehydration processes. The material composition and corresponding sheet names are listed in Table 1.

**Figure 4:**
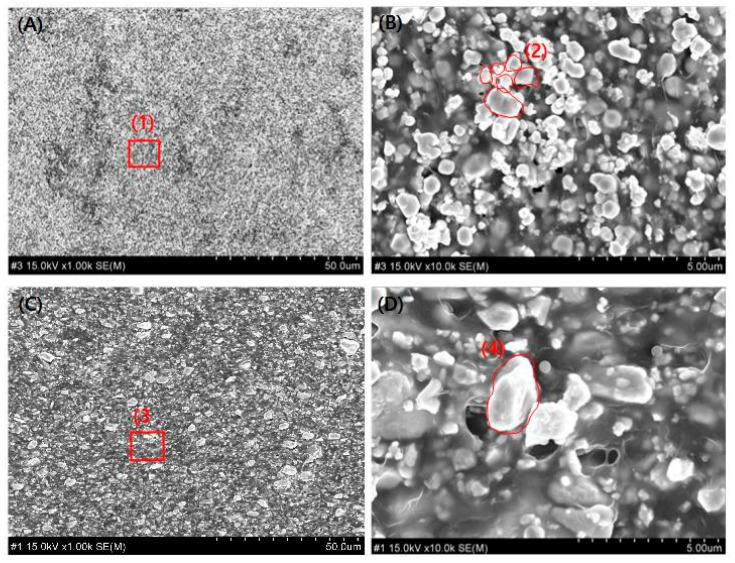
Cross-sectional images of the shielding sheet: (**A**) Image (RS-3) showing a shielding sheet fabricated without sintering the composite material; (**B**) Magnified view of area (1) in (**A**); (**C**) Image showing the sheet fabricated after sintering; (**D**) Magnified view of area (3) in (**C**). In (**B**), area (2) represents the state where particles are clustered before sintering, whereas in (**D**), area (4) shows the grown particles after sintering.

A preliminary comparison and evaluation were conducted by incorporating a sintering process before mixing the shielding sheet materials. Tungsten has a melting point of 3400 °C, whereas bismuth oxide melts at 800 °C. Owing to this significant difference, each material was sintered separately before mixing. The properties of shielding sheets produced with and without the sintering process under identical conditions were compared. Consequently, tungsten was heat-treated at 1250–1600 °C, while bismuth oxide underwent heat treatment at 400–450 °C [44,45]. The particle sizes were approximately 5–20 μm for tungsten and 10–50 μm for bismuth oxide. As shown in Figure 4, particle growth was observed in the sheets after sintering, which is attributed to the coalescence of smaller particles with high surface energy into larger particles [46]. As depicted in Figure 4b,d, tungsten retained smaller particles, whereas bismuth oxide maintained larger particles, leading to polydispersed particle packing. The mixture of large and small particles allows the smaller particles to fill the voids between the larger ones, thereby increasing packing density. By selecting an appropriate ratio, porosity can be minimized. However, as shown in Figure 4, particle agglomeration occurred, resulting in reduced uniformity in particle size. While density and shielding performance could be somewhat improved, large-scale production may suffer from lower dispersion uniformity owing to material agglomeration.

In this study, the shielding properties of standard lead sheets with different thicknesses were presented, as outlined in Table 2, to validate the criteria for evaluating radiation shielding performance. This approach is expected to be effective for assessing and comparing the performance of the fabricated shielding sheets.

The shielding performance and density of the shielding sheets under the same conditions before and after the sintering process were comparatively analyzed, as shown in Table 3. A difference in shielding performance before and after sintering was observed, with a general trend of improved shielding performance after sintering. When the effective energy was 22.5 keV, the shielding performance of RS-3 (90 wt%) improved by approximately 3.12%, and at a slightly higher incident energy of 53.8 keV, it improved by approximately 4%. In general, the higher the tungsten density, the better the shielding effect. The density before and after sintering also improved by approximately 14.88% for RS-3 (90 wt%), indicating that the sintering process enhanced the density of the shielding material, thereby improving shielding performance, as outlined in Table 4.

**Figure 5:**
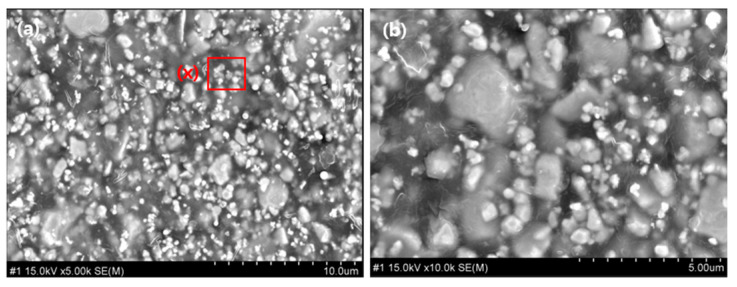
Images of shielding sheets before and after solvent evaporation: (**a**) Image (RS-1) showing the sheet after solvent evaporation; (**b**) Magnified view of area (x) in (**a**).

In addition, the solvent evaporation method was employed while fabricating the shielding sheets to mix the shielding material with the polymer, thereby inducing a reduction in internal voids within the shielding structure. This approach aimed to improve the density of the shielding sheet. By fully evaporating the solvent (THF) used for dissolving polyethylene, the spacing between the shielding material particles was reduced, leading to decreased porosity and increased density. The removal of the solvent occurs during the dehydration process, and it is necessary to increase the concentration of PE to prevent particle rearrangement and reduce agglomeration. Figure 5 shows the images after solvent evaporation, in which bubbles and residual solvents were effectively removed. As outlined in Table 5, the density difference before and after this process resulted in an approximate 13.18% change for RS-3 (90 wt%), indicating no significant improvement.

**Figure 6:**
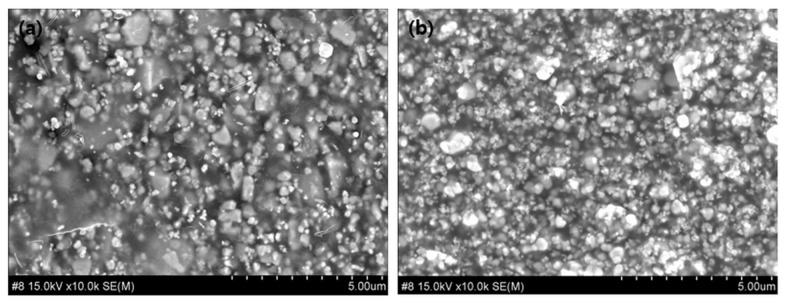
Cross-sectional images of shielding sheets before and after compression: (**a**) Image (RS-1) showing the sheet before compression; (**b**) Image (RS-1) showing the cross-section after compression.

The characteristic changes through compression molding, which is expected to be the most effective external condition for improving density when manufacturing shielding sheets, were observed, as outlined in Table 6 and Table 7. A pressing process was performed by applying forced compression to enhance the density of the fabricated shielding sheet. The process was conducted at 120 °C under a pressure of 45–50 MPa for 540 s, producing a shielding sheet with a thickness of 0.3 ± 0.05 mm. After compression, the thickness was reduced by approximately 0.05–0.06 mm. As shown in Figure 6, the cross-sectional image indicates a slight reduction in the area where PE aggregates, and a more uniform distribution of the shielding material is observed.

The shielding performances before and after the compression process were compared. At an effective energy of 39.4 keV, RS-1 showed an improvement in shielding efficiency of approximately 6.85%. At 48.2 keV, RS-3 demonstrated an enhancement of approximately 9.47%. The fabricated sheet densities also showed significant increases: approximately 14.03% for RS-1, 13.68% for RS-2, and 12.90% for RS-3. These results confirm that compression is a critical processing technique for improving the density of shielding sheets. Table 8 presents the tensile strength results, showing that as the content of the shielding material increased, the shielding performance decreased.

## 4. Discussion

Depending on the type of shielding material, combining it with a polymer to fabricate it into sheet form is advantageous for securing flexibility. However, this method poses challenges in ensuring reproducibility and maintaining consistent shielding performance. As shown in this study, as additional processing techniques are applied, irregular particle arrangements within the shielding sheet become more pronounced. Increasing the density of the material is essential to addressing this issue.

Previous studies have attempted to reduce voids by decreasing particle size; however, nanoparticle fabrication processes are associated with high costs and significant stability concerns [47]. Therefore, polydisperse particle packing, which minimizes the gaps between particles, is considered a more effective approach to increasing density.

**Figure 7:**
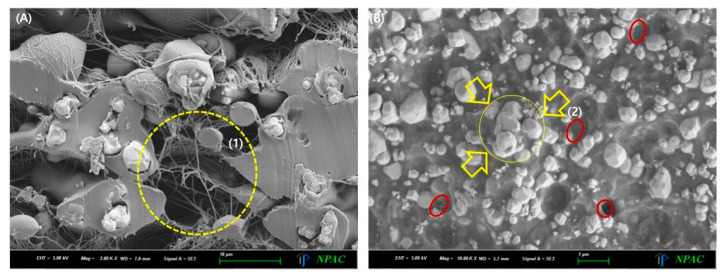
Shielding sheet using nanofibers and nanomaterials: (**A**) Voids (1) formed during the fabrication of tungsten nanofibers; (**B**) Multiple voids (2) observed inside the sheet fabricated using nanosized tungsten particles.

The spacing between particles can vary depending on several factors, such as particle size, shape, and affinity with the polymer, all of which contribute to the formation of voids. Particle size reduction is the most commonly used strategy to reduce these voids. In particular, the nanoparticle process has recently become one of the most widely used methods for improving shielding performance [48]. However, as shown in Figure 7, pinholes inevitably occur during nanofabrication. Therefore, rather than focusing on particle size reduction, this study proposes a method to reduce interparticle spacing by enhancing the affinity between particles and the polymer during fabrication.

As shown in Figure 7b, even in single-material systems, pinholes can occur during the fabrication process. Instead of relying solely on nanoparticle fabrication, uniform dispersion of particles with varying sizes proves to be a more effective approach. Ensuring a uniform distribution of particles across the shielding sheet area can ultimately enhance shielding performance. As demonstrated in the results of this study, particle sintering improves interparticle connectivity, which helps reduce porosity. Furthermore, the solvent used to improve polymer affinity, when combined with active evaporation and final thermal compression molding, minimizes internal voids within the shielding sheet, thereby enhancing overall shielding efficiency. Therefore, as shown in Figure 7b, the processing steps lead to the aggregation of particles by controlling their size. This aggregation enhances the interaction effects, thereby improving the shielding performance.

An increase in the particle-to-volume ratio within the same area leads to higher material density, which in turn improves the radiation attenuation effect. Therefore, rather than focusing solely on reducing particle size as in conventional approaches, improving economically viable processing techniques would be more appropriate. However, insufficient polymer content can impair flexibility, making it necessary to ensure enough polymer matrix through hybrid composite design. As shown in this study, dispersing a mixture of tungsten and bismuth particles in a polymer base can be more effective than using a single material, particularly in reducing internal voids.

The sintering process applied to the particles used in the shielding material can result in a variation of particle sizes; however, it also has the drawback of requiring an additional processing step, thereby increasing the overall production cost. Therefore, as demonstrated in this study, the compaction process is expected to effectively and economically improve the shielding performance.

The shielding sheets used as materials for aprons worn by medical personnel are designed to protect against both primary and scattered radiation. However, a potential risk of scattered radiation originating from the shielding sheet exists in cases where the shielding material comes into direct contact with the body. In particular, at higher energy levels, backscattering may occur, which could lead to certain limitations in the shielding effectiveness against primary radiation [49].

The limitations of this study are as follows: verifying the validity of composite materials by combining tungsten-based metal and bismuth oxide rather than comparing the individual materials can limit the overall understanding of material performance. In addition, the study does not include a comparison with other processing technologies. For future practical applications, we aim to compare the study results with those obtained from nanofabrication processes and to evaluate the economic feasibility and effectiveness of mass-produced shielding sheets.

In medical radiation shielding, materials are commonly fabricated in the form of protective garments, which require flexibility for diverse applications and reproducibility of consistent shielding performance. Enhancing the technical aspects of the manufacturing process is crucial to achieving this.

Therefore, the effective utilization of processes such as sintering, solvent evaporation, and compression can significantly contribute to improved shielding performance. In particular, the thermal press molding process significantly contributed to increasing the material density and enhancing the shielding performance. As such, this process is considered one of the most effective methods for ensuring reproducibility.

## 5. Conclusions

This study investigated an alternative approach to the commonly used nanoparticle-based process for enhancing the performance of shielding sheets used in medical radiation-protective garments. A method for effectively increasing the internal density of shielding materials was proposed. In particular, the polydisperse particle packing technique, which reduces interparticle spacing during the fabrication process, was found to have a positive impact on improving the shielding performance. In particular, in the mixing process of composite materials, post-sintering mixing resulted in an approximately 4% improvement in shielding performance. In the thermal press compaction process, an enhancement of about 10% in shielding performance was verified, corresponding to 0.25 mmPb in terms of lead equivalence. Therefore, the sintering process of the shielding material particles, the removal of the polymer solvent during the mixing process, and the thermal press compression of the final shielding body contributed to improving the control of the particle spacing. These processes increased the overall density of the final shielding material, thereby enhancing its shielding performance. This study confirms that optimizing density enhancement strategies for radiation shielding materials can lead to significant improvements in shielding performance.

## Figures and Tables

**Table 1:** Composition ratios of composite materials.

Sheet Name	W	Bi_2_O_3_	PE
RS-1	80	10	10
RS-2	85	10	5
RS-3	90	5	5

**Table 2:** Evaluation of the shielding performance of standard lead sheets (0.1 mm, 0.2 mm, and 0.3 mm).

Radiation Type	Effective X-Ray Energy (keV)	Lead Shielding Rate (%)		
		Thickness (mmPb)		
		0.1 mm	0.2 mm	0.3 mm
X-ray	22.5	84.24	98.14	100
	24.3	83.53	95.07	98.12
	30.2	72.78	87.78	93.61
	46.5	63.97	80.43	87.93
	53.8	61.40	78.22	86.41

The standard lead sheets used for the tests were made of lead with a purity of 99.99% or higher.

**Table 3:** Performance of shielding sheets fabricated before and after sintering of shielding materials.

Radiation Type	Effective X-Ray Energy (keV)	Shielding Rate (%)					
		Before Sintering			After Sintering		
		RS-1	RS-2	RS-3	RS-1	RS-2	RS-3
X-ray	22.5	78.41	86.21	92.85	84.74	90.58	95.75
	24.3	73.23	81.85	88.52	81.47	85.78	93.45
	30.2	71.45	77.75	84.41	76.52	82.41	87.89
	46.5	64.44	74.21	78.17	74.14	78.12	81.36
	53.8	60.86	70.32	76.11	68.56	71.11	79.12

**Table 4:** Density measurement results of the shielding sheets before and after sintering.

	Density (g/cm^3^)					
	Before Sintering			After Sintering		
	RS-1	RS-2	RS-3	RS-1	RS-2	RS-3
Mean	13.11	13.12	13.24	14.88	14.97	15.21
Standard Deviation	0.07	0.06	0.16	0.15	0.18	0.21
Coefficient of Variation (%) *	0.53	0.46	1.21	1.01	12.01	1.38

* Standard deviation/mean value × 100.

**Table 5:** Density measurement results of the shielding sheets after solvent evaporation.

	Density (g/cm^3^)					
	Before Solvent Evaporation			After Solvent Evaporation		
	RS-1	RS-2	RS-3	RS-1	RS-2	RS-3
Mean	12.25	12.84	13.20	13.98	14.21	14.94
Standard Deviation	0.03	0.02	0.01	0.02	0.16	0.18
Coefficient of Variation (%) *	0.24	0.15	0.08	0.14	11.26	12.05

* Standard deviation/mean value × 100.

**Table 6:** Comparison of the shielding performance of the sheets before and after compression in the shielding sheet fabrication process.

Radiation Type	Effective X-Ray Energy (keV)	Shielding Rate (%)					
		Before Compression			After Compression		
		RS-1	RS-2	RS-3	RS-1	RS-2	RS-3
X-ray	24.1	78.91	86.41	94.12	82.78	90.52	96.45
	26.8	74.92	82.21	90.45	77.26	88.12	94.23
	39.4	70.63	76.74	86.87	75.47	84.47	89.36
	48.2	68.32	71.56	77.23	70.18	81.58	84.54
	54.4	62.77	66.28	74.74	68.63	78.23	82.11

**Table 7:** Density measurements of the shielding sheets before and after the compression process.

	Density (g/cm^3^)					
	Before Compression			After Compression		
	RS-1	RS-2	RS-3	RS-1	RS-2	RS-3
Mean	13.11	13.23	13.49	14.95	15.04	15.23
Standard Deviation	0.05	0.04	0.05	0.15	0.18	0.20
Coefficient of Variation (%) *	0.38	0.30	0.37	1.00	1.20	1.31

* Standard deviation/mean value × 100.

**Table 8:** Tensile strength measurements of shielding sheets before and after the compaction process.

	Tensile Strength (MPa)					
	Before Compression			After Compression		
	RS-1	RS-2	RS-3	RS-1	RS-2	RS-3
Mean	18.5	16.5	15.8	18.4	17.1	15.5
Standard Deviation	0.03	0.03	0.04	0.05	0.13	0.16
Coefficient of Variation (%) *	0.16	0.18	0.25	0.27	0.76	1.03

* Standard deviation/mean value × 100.

## Data Availability

All data generated during this study are included in the manuscript.

## Abbreviations

The following abbreviations are used in this manuscript:

FESEM	Field emission scanning electron microscope
HVL	High-value layer
RPE	Radiation protection efficiency
